# Value of information methods to design a clinical trial in a small population to optimise a health economic utility function

**DOI:** 10.1186/s12874-018-0475-0

**Published:** 2018-02-08

**Authors:** Michael Pearce, Siew Wan Hee, Jason Madan, Martin Posch, Simon Day, Frank Miller, Sarah Zohar, Nigel Stallard

**Affiliations:** 1 Complexity Science, University of Warwick, Coventry, UK; 2 Statistics and Epidemiology, Division of Health Sciences, Warwick Medical School, University of Warwick, Coventry, UK; 3 Warwick Clinical Trials Unit, Division of Health Sciences, Warwick Medical School, University of Warwick, Coventry, UK; 4 Section of Medical Statistics, CeMSIIS, Medical University of Vienna, Vienna, Austria; 5 Clinical Trials Consulting and Training Limited, Buckingham, UK; 6 Department of Statistics, Stockholm University, Stockholm, Sweden; 7 INSERM, U1138, team 22, Centre de Recherche des Cordeliers, Université Paris 5, Université Paris 6, Paris, France

**Keywords:** Decision theory, Health economics, Power, Rare disease, Regulator, Type I error, Value of information

## Abstract

**Background:**

Most confirmatory randomised controlled clinical trials (RCTs) are designed with specified power, usually 80% or 90%, for a hypothesis test conducted at a given significance level, usually 2.5% for a one-sided test. Approval of the experimental treatment by regulatory agencies is then based on the result of such a significance test with other information to balance the risk of adverse events against the benefit of the treatment to future patients. In the setting of a rare disease, recruiting sufficient patients to achieve conventional error rates for clinically reasonable effect sizes may be infeasible, suggesting that the decision-making process should reflect the size of the target population.

**Methods:**

We considered the use of a decision-theoretic value of information (VOI) method to obtain the optimal sample size and significance level for confirmatory RCTs in a range of settings. We assume the decision maker represents society. For simplicity we assume the primary endpoint to be normally distributed with unknown mean following some normal prior distribution representing information on the anticipated effectiveness of the therapy available before the trial. The method is illustrated by an application in an RCT in haemophilia A. We explicitly specify the utility in terms of improvement in primary outcome and compare this with the costs of treating patients, both financial and in terms of potential harm, during the trial and in the future.

**Results:**

The optimal sample size for the clinical trial decreases as the size of the population decreases. For non-zero cost of treating future patients, either monetary or in terms of potential harmful effects, stronger evidence is required for approval as the population size increases, though this is not the case if the costs of treating future patients are ignored.

**Conclusions:**

Decision-theoretic VOI methods offer a flexible approach with both type I error rate and power (or equivalently trial sample size) depending on the size of the future population for whom the treatment under investigation is intended. This might be particularly suitable for small populations when there is considerable information about the patient population.

**Electronic supplementary material:**

The online version of this article (10.1186/s12874-018-0475-0) contains supplementary material, which is available to authorized users.

## Background

Prior to approval a drug typically goes through various phases of clinical development, beginning with assessing pharmacology in humans (phase I), followed by exploration of therapeutic efficacy (phase II) and finally, confirmation of the effectiveness (phase III). This is not a necessary ordering, for example, prior to presenting overall clinical development, results and issues of the drug’s efficacy and safety (this list is long) to regulatory agencies, further investigation of the effect on human pharmacology may be conducted. Based on the submitted information, the regulatory authorities approve the product that has demonstrated safety and effectiveness for the intended population.

Treatments for rare diseases may not go through all the phases of clinical development prior to submission to regulatory authorities. Buckley presented a brief summary of treatments intended for rare diseases and approved by European regulator that did not go through all these phases [1]. The difference is usually attributable to the small population where it is infeasible or impossible to recruit many patients for trials.

The focus of this paper is the design of phase III trials in particular in a small population. One of the fundamental issues in clinical trial design is sample size determination. The most common approach is to perform a power calculation for a hypothesis test. In a two-arm randomised controlled trial aiming to establish superiority of an experimental treatment to the standard treatment, the null hypothesis may state that the experimental treatment is not superior to the control.

The sample size is determined by restricting type I and II error rates. A type I error occurs when a true null hypothesis is rejected and a type II error occurs when a false null hypothesis is not rejected. Typically, the one-sided type I error rate, *α*, is set to 0.025. The type II error rate, *β*, is set to 0.10 or 0.20 for some specified alternative hypothesis. Correspondingly, the power to detect the predefined value, 1−*β*, is 0.90 or 0.80. Values for *α* and *β* are customarily set without consideration of the severity or prevalence of the disease. In practice, however, clinical trials in rare diseases have smaller sample sizes than those in more common conditions, indicating that conventional type I and type II error rates are being compromised in this setting [2, 3]. Increasing the type I error rate might be appropriate in a trial for rare disease as the small population means that the number of patients who would benefit from an effective treatment may be small and so it may be justifiable to have alternate levels of type I error so that an effective treatment may be made available more easily.

To determine the sample size it seems reasonable to consider information such as previous trial outcomes, the cost of making type I or type II errors, the number of people affected, the financial burden of the treatment and other costs and rewards that will result from the treatment being approved and marketed. One model that could be used to optimise the sample size accounting for all the information is the Bayesian decision theoretic approach. In this framework, decisions are made so as to maximise a utility function that quantifies their desirability of an outcome. The utility function may be made up of observed responses from a sample of patients, the costs of treating patients and conducting the trial, and the profits from a successful treatment. Some authors have proposed to include the cost of trials, the profit from a successful trial (or a loss from an unsuccessful trial), two endpoints (e.g. efficacy and adverse events) and the size of the population, *N*, so that the sample size required for the trial is optimised (see Hee et al. [4]). By including *N* we also incorporate the potential gain to future patients. Such an approach seems particularly suitable in the setting of a small population, when there is likely to be considerable knowledge of the size of the target population prior to the start of a clinical trial and this is likely to be much smaller than in other settings.

## Methods

### A Bayesian decision theoretic approach to sample size determination

In our approach we assume that following safety and efficacy exploration studies, once the drug has been shown to be effective in a phase III trial for the intended population, it obtains regulatory approval. The objective of a phase III trial is primarily to confirm effectiveness. The typical design considered to be the gold standard in providing the best evidence in assessing efficacy is the randomised controlled trial (RCT). Let *n* be the size of the RCT and assume that patients are randomised in a 1:1 ratio to either the experimental or standard arm. Let **Y**_*i*_=(*Y*_*i*1_,…,*Y*_*in*/2_), *i*=(*E*,*S*), denote the outcomes and $\bar {Y}_{i}$ denote the sample mean from *n*/2 patients from the experimental (E) and standard treatment (S) arms, respectively. Assume $\bar {Y}_{i}$ is normally distributed with mean *θ*_*i*_ and known variance, $2\sigma _{i}^{2}/n$, that is $\bar {Y}_{i}\sim N\left (\theta _{i}, 2\sigma _{i}^{2}/n\right)$. In the classical frequentist setting, we test the null hypothesis that the two population means, *θ*_*E*_ and *θ*_*S*_, are equal, equivalently *θ*=*θ*_*E*_−*θ*_*S*_=0. The difference of the sample means, denoted by $\bar {X} = \bar {Y}_{E} - \bar {Y}_{S}$ is normally distributed with mean *θ* and variance *τ*^2^/*n* where *τ*^2^/*n*=4*σ*^2^/*n* when $\sigma _{E}^{2}=\sigma _{S}^{2}=\sigma ^{2}$ (equal variance) or $\tau ^{2}/n = 2\left (\sigma _{E}^{2} + \sigma _{S}^{2}\right)/n$ when $\sigma _{E}^{2} \ne \sigma _{S}^{2}$ (unequal variance).

In the Bayesian setting, *θ* is assumed to be unknown but to follow a distribution with specified form. Assume that the prior density of *θ*, which measures the belief regarding the parameter prior to observing any responses, is $N\left (\mu _{0}, \sigma _{0}^{2}\right)$. The prior density may be elicited from previous trials, case studies or experts’ opinions [5–9]. Following observations from patients, the prior is updated to give a posterior distribution summarising belief about *θ* given the observed data.

In the Bayesian decision theoretic framework, a utility function summarises the value of all possible actions given *θ*. The action that maximises the expectated value of this utility over the posterior distribution of *θ* given the observed data can then be chosen as the optimal action. As with frequentist hypothesis testing, we may consider that at the end of the trial, there are two possible actions, to reject the null hypothesis or not. The utility function depends on the sample size *n*, and the decision, *d*∈{do not reject *H*_0_, reject *H*_0_}, given *θ*. Let $d(\bar {x}, n)$ denote the action taken at the end of the trial given data $\bar {x}$ with a sample size *n* and *G*(*n*,*d*,*θ*) denote the utility function. We may also choose *n* optimally. As we will not have observed any responses during the planning stage, the expected utility is obtained from the distribution of $\bar {x}$ given *θ* and *n*. As *θ* is unknown, the expectation of the expected utility is taken over the prior density [4]. The expected utility is then a function of the sample size and decision rule, denoted by

1 $$ \mathcal{G}(n,d) = \int_{\bar{x}} \int_{\Theta} G(n,d(\bar{x}, n),\theta) f(\theta|\bar{x},n) \ d\theta f(\bar{x}|n) \ d\bar{x},   $$

where $f(\theta |\bar {x},n)$ is the posterior density of *θ* given $\bar {x}$ and

$$f(\bar{x}|n) = \frac{1}{\sigma_{x}} \phi\left(\frac{\bar{x} - \mu_{0}}{\sigma_{x}} \right) $$

is the prior-predictive density function for $\bar {x}$ before sampling, with $\sigma _{x}^{2} = \sigma _{0}^{2} + \tau ^{2}/n$ the prior predictive variance of $\bar {x}$ and *ϕ*(·) the normal density function. If the action taken at the end of the trial is determined by the outcome of some hypothesis test, the function *d* and hence the expected utility $\mathcal {G}(n,d)$, will depend on the type I error rate, or equivalently the critical value, for that test, as described in more detail in the next subsection. The optimisation problem then becomes one of choosing the trial sample size and test type I error rate.

### Formulation of utility function

We assume that the decision maker for our proposed model represents society. A reward corresponds to improved treatment either of patients in the trial or of future patients if the experimental treatment receives regulatory approval.

The regulator is assumed to approve the experimental treatment if the observed difference, $\bar {X}=\bar {x}$, is greater than a threshold, $z_{\alpha }\tau /\sqrt {n}$, where *z*_*α*_ is the upper *α* percentile of the standard normal distribution, so that $d(\bar {x},n)= $ ‘reject *H*_0_’ or ‘do not reject *H*_0_’ for $\bar {x}$ above or below this threshold. This is equivalent to a classical frequentist analysis of the primary endpoint using a significance test conducted at (one-sided) level *α*. This assumption is similar to that proposed by Pezeshk et al. who also assume that the regulatory agency will test the null hypothesis that there is no difference between the outcomes means at the *α* test size [10]. Since the decision function *d* then depends only on *z*_*α*_, we will write $\mathcal {G}(n,z_{\alpha })$ for $\mathcal {G}(n,d)$ given by (1) above, and refer to *z*_*α*_ as the significance level threshold.

We assume that the size of the population for the treatments we are testing is known, and denote this by *N*. The number of patients that can be treated following the trial depends on *N* and on the trial sample size. We assume that during the trial a proportion *ρ* of patients are enrolled in the trial, so that there are *n*(1−*ρ*)/*ρ* concurrent patients not enrolled in the trial and the number of patients remaining to be treated is *N*−*n*/*ρ*.

We suppose that the utility given $\bar {x}$ represents the gain from treating patients with the experimental treatment. There are *n*/2 such patients in the trial and, if $\bar {x} \geq z_{\alpha }\tau /\sqrt {n}$ so that the new treatment is approved, *N*−*n*/*ρ* such patients following the trial. The reward for each treated patient is taken proportional to the true effect size, i.e. the more effective the treatment is the greater the reward will be. Note that it is considered a loss if the treatment effect is < 0.

The utility function also includes fixed and variable financial costs [11]. Fixed costs are incurred regardless of the size of the trial, denoted by *c*_*f*_; these may be the setting up and running cost of the trial. Variable costs depend on the size of the trial. The cost per patient, denoted by *c*_1_, may be administrative costs to recruit, screen, treat and follow-up patients in the conduct of the trial. Our proposed utility function also includes additional costs, either monetary or harmful effects, of treating a patient with the experimental treatment, denoted by *c*_2_. All these costs may be scaled to one unit of efficacy such that they are relative to *θ*. Alternatively, the treatment efficacy may be scaled to one unit of cost such that it is relative to monetary reward and costs [12].

The utility at the end of the trial if the regulator approves the experimental treatment is the reward from treating patients in the trial and the future less the cost of treating them and both the fixed and variable trial costs. If the regulator does not approve the experimental treatment, the utility is the reward from treating patients in the trial minus the cost of treating patients in the trial with the experimental treatment and fixed and variable costs. Possible actions are to approve or not the experimental treatment. Thus, the utility function, taken relative to the baseline of treating all patients with the control treatment, is

2 $$ G(n,d, \theta) = \left\{ \begin{array}{l} (\theta - c_{2})\left(N - \frac{n}{\rho}+\frac{n}{2}\right) - c_{1}n \\ \quad - c_{f}I_{\{n>0\}} \quad d = \text{reject}\ H_{0}, \\ (\theta - c_{2})\frac{n}{2} - c_{1}n \\ \quad - c_{f}I_{\{n>0\}} \quad d = \text{do not reject}\ H_{0}, \end{array} \right.   $$

where *I*_{*n*>0}_=1 if *n*>0 and 0 otherwise. The expected utility given by (1) is then

3 $$ {\begin{aligned} & \mathcal{G}(n,z_{\alpha}) \\ & \qquad \qquad= \iint \left\{ (N - n/\rho)(\theta - c_{2})I_{\{\bar{x} \geq z_{\alpha}\tau/\sqrt{n}\}} \right.\\ & \quad\quad\quad\quad\quad \left.+ \frac{n}{2}(\theta - c_{2}) - c_{1}n - c_{f}I_{\{n>0\}} \right\} \\ & \quad\quad\quad\quad\quad\times f(\theta|\bar{x}) f(\bar{x}|n) \ d\theta \ d\bar{x} \\ & \qquad \qquad= (N - n/\rho) \left\{(\mu_{0} - c_{2})\Phi(-Z) + \frac{\sigma_{0}^{2}}{\sigma_{x}} \phi(Z) \right\}\\ & \quad\qquad \qquad+ \frac{n}{2}(\mu_{0} - c_{2}) - c_{1}n - c_{f}I_{\{n>0\}},  \end{aligned}}  $$

where *Φ*(·) is the normal cumulative distribution function and $Z = (z_{\alpha }\tau /\sqrt {n} - \mu _{0})/\sigma _{x}$ is the z-score of the significance level threshold, *z*_*α*_, on the prior predictive distribution of $\bar {x}$. The full derivation is presented in the Additional file 1.

### Optimisation

The optimal sample size and significance level threshold are obtained by maximising the expected utility, $\mathcal {G}(n,z_{\alpha })$. Let $z_{\alpha }^{*}$ denote the optimal value for the significance level threshold. This is obtained for any *n* by differentiating $\mathcal {G}(n,z_{\alpha })$ with respect to *z*_*α*_, to obtain

4 $$ {\begin{aligned} \frac{d}{dz_{\alpha}}\mathcal{G}(n, z_{\alpha}) &= (N - n/\rho)(\mu_{0} - c_{2})\frac{d}{dz_{\alpha}}\Phi(-Z)\\ & \quad + (N - n/\rho)\frac{\sigma_{0}^{2}}{\sigma_{x}}\frac{d}{dz_{\alpha}}\phi(Z) \\ & = (N - n/\rho) \left[{\vphantom{\frac{\sigma_{0}^{2}}{\sigma_{x}}\frac{\tau(z_{\alpha}\tau/\sqrt{n} - \mu_{0})/\sqrt{n}}{\sigma_{x}^{2}} \phi(Z)}}(\mu_{0} - c_{2})\frac{\tau/\sqrt{n}}{\sigma_{x}} \phi(Z) \right.\\ & \quad \left.+ \frac{\sigma_{0}^{2}}{\sigma_{x}}\frac{\tau \left(z_{\alpha}\tau/\sqrt{n} - \mu_{0} \right)/\sqrt{n}}{\sigma_{x}^{2}} \phi(Z) \right].  \end{aligned}}  $$

Equating the differentiated expression (4) to zero, for a given sample size *n* the optimal *z*_*α*_ is

5 $$ z_{\alpha}^{*} = c_{2}\frac{\sigma_{x}^{2}\sqrt{n}}{\sigma_{0}^{2}\tau} - \mu_{0}\frac{\tau}{\sigma_{0}^{2}\sqrt{n}}.  $$

Subsequently, the optimal sample size, *n*^∗^, is obtained numerically by substituting (5) into (3) (see Additional file 1 for details).

## Results

### Application to a case study

Abrahamyan et al. presented a decision analytic value of information (VOI) model for assessing evidence on treatments for children with severe haemophilia A, a rare disease [12]. They summarised that there are three types of treatments currently available in various developed countries; alternate day prophylaxis (AP), on-demand (OD) and tailored prophylaxis (TP) of intravenous administration of recombinant Factor VIII (FVIII). They utilised information from US and Canada studies to evaluate whether or not to conduct another trial. They performed three pairwise comparisons; TP vs. AP, OD vs. TP and OD vs. AP, and in each comparison the optimal decision, whether to conduct another trial or to accept one of the treatments as a standard therapy, was given depending on the maximum acceptable price per unit health gain.

We adapted this work to illustrate the application of our proposed model. Table 1 shows the estimated values used in our example. The primary endpoint was binary, whether or not the patient had magnetic resonance imaging (MRI)-detected joint damage. Similar to Abrahamyan et al., we assume large sample approximations to this binary endpoint and so the efficacy mean, $\hat {\mu }_{t}, t=(\text {AP}, \text {OD}, \text {TP})$, is the proportion of patients without MRI-detected joint damage. The sample and prior variances are $\hat {\sigma }^{2}_{t} = \hat {\mu }_{t}(1-\hat {\mu }_{t})$ and $\hat {\sigma }_{0t}^{2}=\text {var}(\hat {\mu }_{t})=\hat {\mu }_{t}(1-\hat {\mu }_{t})/n_{0t}$, respectively where *n*_0*t*_ is the number of prior observations for treatment *t* reported by Abrahamyan et al., that is *n*_0,AP_=27,*n*_0,OD_=29 and *n*_0,TP_=24.

**Table 1:** Summary statistics and costs (in dollar, $) by treatment for haemophilia A, adapted from Abrahamyan et al. [12]

	Treatment, *t*		
Statistics	AP	OD	TP
Prior mean, $\hat {\mu }_{t}$	0.9259	0.5517	0.7917
Prior variance, $\hat {\sigma }^{2}_{0t}$	0.0025	0.0085	0.0069
Sample variance, $\hat {\sigma }^{2}_{t}$	0.0686	0.2473	0.1649
Mean cost, *c*_2*t*_ ($)	176,397	56,619	117,651

AP, Alternate day prophylaxis; OD, on-demand; TP, tailored prophylaxis

To illustrate our model, consider a two-arm RCT comparing the on-demand (OD), which we assumed to be the standard treatment, with the tailored prophylaxis (TP). The point prevalence of haemophilia A in US is 7.0 per 100,000 which is about 22,400 in the US given the US population of approximately 320 million [13, 14].

Let the measure of efficacy be the absence of MRI-detected haemophiliac joint damage and the unknown parameter measuring treatment benefit be the difference of proportion of patients without MRI-detected joint damage valued in monetary terms using the value the decision-maker places on each unity of efficacy, which will be denoted *λ*. The prior distribution for the unknown parameter is then normal with prior mean *μ*_0_=*λ*(*μ*_TP_−*μ*_OD_) and prior variance $\sigma ^{2}_{0}=\lambda ^{2}\left (\sigma ^{2}_{0,\text {TP}}+\sigma ^{2}_{0,\text {OD}}\right)$. As shown in Table 1, $\sigma _{TP}^{2} \neq \sigma _{OD}^{2}$, therefore, the sample variance is estimated by $\tau ^{2}=2\lambda ^{2}\left (\sigma ^{2}_{\text {TP}}+\sigma ^{2}_{\text {OD}}\right)$. Following Abrahamyan et al., take *λ*=*$*400,000 leading to the values given in Table 2. Suppose the fixed financial cost incurred from conducting the trial is *c*_*f*_=*$*1m, the cost of conducting the trial per patient is *c*_1_=*$*5,000 and the cost of treating a patient is *c*_2_=*c*_2,TP_−*c*_2,OD_=*$*61,032 (see, Table 2).

**Table 2:** Parameter estimates for the pairwise comparison between on-demand (OD) and tailored prophylaxis (TP)

Parameter	Estimates
Population size, *N*	4000
Prior mean, *μ*_0_=*λ*(*μ*_TP_−*μ*_OD_), ($)	96000
Prior variance, $\sigma _{0}^{2}=\lambda ^{2}\left (\sigma ^{2}_{0,\text {TP}}+\sigma ^{2}_{0,\text {OD}}\right)$, ($)	(49638)^2^
Sample variance, $\tau ^{2}=2\lambda ^{2}\left (\sigma ^{2}_{\text {TP}}+\sigma ^{2}_{\text {OD}}\right)$, ($)	(363202)^2^
Cost of conducting the trial per patient, *c*_1_, ($)	5000
Cost of treating a patient, *c*_2_=*c*_2,TP_−*c*_2,OD_, ($)	61032
Fixed financial cost incurred from conducting the trial, *c*_*f*_, ($)	1 million

Similar to Abrahamyan et al., we assume annual haemophilia A incidence of 200 with 1/5 of patients recruited to the trial (*ρ*=0.2). Let the time horizon be 20 years and assume that all new cases will be prescribed with the recommended treatment after the trial. The total number of future patients who will benefit from the new treatment is thus 4000−5*n*.

**Fig. 1:**
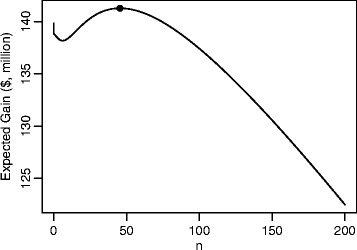
Expected utility, $\mathcal {G}\left (n,z_{\alpha }^{*}\right)$, against *n*

Figure 1 shows the expected utility for $z_{\alpha } = z^{*}_{\alpha }$ for a range of values of *n*. The optimal sample size, corresponding to the value for which the expected utility is highest is shown by the plotted point and is equal to *n*^∗^=46 (i.e. 23 patients per arm), $\mathcal {G}\left (n^{*},z_{\alpha }^{*}\right)=\$141 \text {million and the threshold to approve TP is} z_{\alpha }^{*}=0.36876, \text {equivalent to} \alpha =0.35615$.

Both *n*^∗^ and $z_{\alpha }^{*}$ are far from their conventional values. In particular, except in the cases when *n*=0, the power is much smaller than a conventional level. Taking *α*=0.025 and *β*=0.8 for an alternative *θ*_*A*_=*σ*_0_/2=*$*24,819 would require *n*=268. The expected utility for this design is $109 million.

### Operating Characteristics

**Fig. 2:**
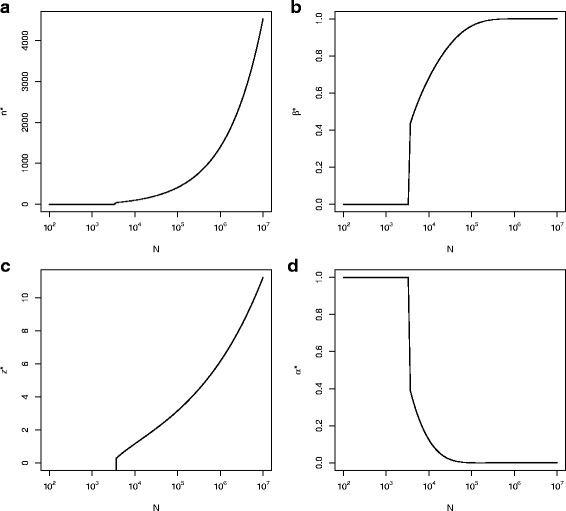
Optimal (**a**) sample size, *n*^∗^, (**b**) type II error rate, *β*^∗^, in order to detect an alternative *θ*_*A*_=*σ*_0_/2=*$*24819 (**c**) $z_{\alpha }^{*}$ and (**d**) type I error rate, *α*^∗^ against the size of the population, *N*, with fixed $\mu _{0}=\$96000,\sigma _{0}^{2}=(\$49638)^{2}$, *τ*^2^=(*$*363202)^2^, *c*_1_=*$*5000,*c*_2_=*$*61032 and *c*_*f*_=*$*1 million

Figure 2 shows optimal sample sizes and significance levels obtained from our model for population sizes between 100 and 10,000,000 with other parameters fixed as *θ*∼*N*(96,000,(49,638)^2^),*τ*^2^=(*$*363,202)^2^,*c*_1_=*$*5,000,*c*_2_=*$*61,032 and *c*_*f*_=*$*1m. Figure 2a shows that if the population is small (*N*<3000), the optimal sample size is *n*^∗^=0. In this case the optimal significance level threshold to approve TP is $z_{\alpha }^{*} \rightarrow -\infty $ (equivalently, *α*→1, see Fig. 2c and d respectively), corresponding to an optimal decision to approving the experimental treatment based on the prior belief alone.

Other than for very small population sizes (*N*>3,000) the optimal trial sample size increases with *N*, with log(*n*^∗^) increasing approximately linearly with log(*N*). This reflects work by Cheng et al. and Stallard et al. showing that as *N* increases $n^{*} \propto \sqrt {N}$ [15, 16]. The optimal significance level decreases as *N* increases (Fig. 2d), requiring a stricter level of statistical significance. Figure 2b shows the type II error rate of the test using sample size *n*^∗^ and significance level threshold $z^{*}_{\alpha }$, to detect an alternative *θ*_*A*_=*σ*_0_/2=*$*24,819. In this case the type II error rate increases to approach 1 as *N* increases, so that that power approaches 0. This is reasonable since as *θ*_*A*_<*c*_2_, this true effect difference is insufficient to justify recommendation of the treatment for use in future patients, though is very different to a more conventional approach in which the power will increase with increasing *n*.

**Fig. 3:**
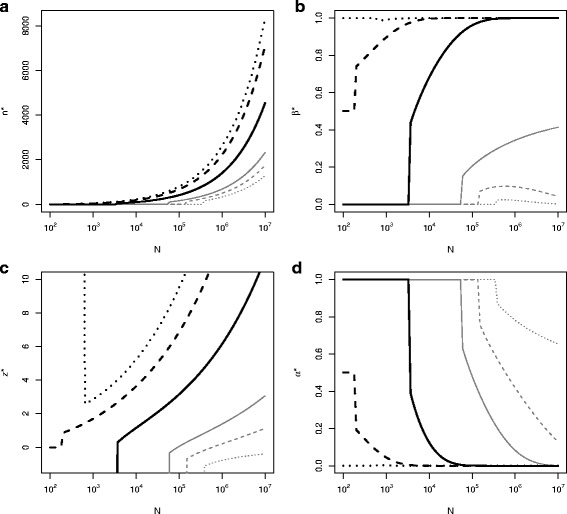
Optimal (**a**) sample size, *n*^∗^, (**b**) type II error rate, *β*^∗^, (**c**) $z_{\alpha }^{*}$ and (**d**) type I error rate, *α*^∗^ in order to detect an alternative *θ*_*A*_=*$*24819 against the size of the population, *N*, with fixed $\mu _{0}=\$96000,\sigma _{0}^{2}=(\$49638)^{2}, \tau ^{2}=(\$363202)^{2}, c_{1}=\$5000$ and *c*_*f*_=*$*1 million for different values of; *c*_2_=0 (light grey dotted line), *c*_2_=*σ*_0_/4=*$*12409 (light grey dashed line), *c*_2_=*σ*_0_/2=*$*24819 (light grey solid line), *c*_2_=*$*61032 (heavy black solid line), *c*_2_=*μ*_0_=*$*96000 (heavy black dashed line) and *c*_2_=*σ*_0_/2+*μ*_0_=*$*120819 (heavy black dotted line)

Figure 3 shows optimal sample size (*n*^∗^), threshold $\left (z_{\alpha }^{*}\right)$, type I (*α*^∗^), and II (*β*^∗^) error rates, against the size of the population for various values of cost, *c*_2_=0,*c*_2_=*$*12,409,*c*_2_=*$*24,819,*c*_2_=*$*61,032,*c*_2_=*$*96,000 and *c*_2_=*$*120,819. Cases include *c*_2_ equal to, above and below *θ*_*A*_ and the prior mean.

When *c*_2_=0 we have *n*^∗^>0 only if *N*>390,000 (Fig. 3a). If *N*<390,000 the optimal decision is to approve the experimental treatment without a trial. When *c*_2_=*μ*_0_, it is optimal not to start the trial if the population size, *N*<200. In this case prior belief is that the expected utility from the new and standard treatments are equal so that rejection or non-rejection of *H*_0_ lead to the same expected utility so *z*_*α*_ can be taken to be *∞* or −*∞*, or equivalently *α* may be taken to be 1 or 0. When *c*_2_>*μ*_0_ although it is optimal to conduct no trial if the population size is less than 640 it is optimal not to approve TP but to continue with the standard treatment, OD. This is shown in Fig. 3d where the optimal type I error rate, *α*^∗^→0. The optimal type II error rate, *β*^∗^ approaches 1 (power approaches 0) as *N* and *n*^∗^ become large when *c*_2_>*θ*_*A*_ and, though this is hard to see from the plot, approaches 0 (power approaches 1) as *N* becomes large when *c*_2_<*θ*_*A*_. Although not shown on these plots, as *c*_2_ becomes very large, the optimal sample size, *n*^∗^, decreases, with *n*^∗^=0 for largest *c*_2_ values reflecting very strong prior belief that the TP is not sufficiently promising to overcome the cost *c*_2_.

Additional calculations were performed to evaluate the sensitivity of the results obtained to specification of the prior mean, *μ*_0_, and variance, $\sigma _{0}^{2}$. Figures illustrating the optimal design parameters, *n*^∗^ and *α*^∗^ are given in the Additional file 2. These show that increasing or decreasing *μ*_0_ has a similar effect on the choice of *n* and *α* as decreasing or increasing *c*_2_ and that increasing or decreasing the prior variance, that is decreasing or increasing the level of prior information assumed, decreases or increases the value of *N* below which *n*^∗^=0.

## Discussion

The methodology of decision theory is the foundation of the value of information (VOI) method presented in the health economics literature. The method is used to assess uncertainty in existing evidence and aid decision-making as to whether to adopt one of the competing treatments or to obtain more information. The decision-theoretic VOI method is based on expected utility maximisation and does not involve constructing and testing of hypotheses. Rather costs of gathering additional information are compared with expected returns in terms of the reduced likelihood of making the wrong decision, and the utility loss from doing so, though the result remains a decision as to whether or not the new treatment should be used and so is analogous to a hypothesis test. This requires specifying a utility function, which will be based on all outcomes (e.g. depending on the decision-maker, this may include health care use, adverse events, quality of life). The underlying methods are consistent in that for reasonable utility functions the decision to approve a treatment will be made so long as it appears sufficiently promising, corresponding to a hypothesis test conducted at some significance level. A number of authors have proposed decision-theoretic approaches to clinical trial design, both in the setting of normally distributed outcomes as considered here and binary outcomes [4]. A distinctive feature of this work is the specific focus on the small population setting. This is reflected in the construction of the utility function used, for example in the assumption of a fixed finite future population, with this decreasing in size depending on the length of the clinical trial.

Regulatory agencies may prefer the use of classical frequentist methods in the evaluation of pharmaceutical products. In the design of a clinical trial based on the frequentist method, the type I error rate, *α*, is usually restricted at a one-sided 0.025 level. This value is a conventional, but arbitrary, choice, and there is some indication that in practice there may be some flexibility depending on severity and/or prevalence of the disease. Our approach involves calculating a z-score for a primary clinical outcome that corresponds to maximising expected utility. It therefore allows a hypothesis testing framework to be maintained, but ensures that the error rate for that hypothesis test on the primary outcome is consistent with a utility maximisation framework. Results from Fig. 3 suggest that different *α*-levels are appropriate for diseases with different prevalence rates. Discussion of choice of *α*-level depending on prevalence and severity of disease has been considered by Montazerhodjat and Lo (2015) [17]. In their working paper they also show that the optimal critical value increases with disease prevalence, but find a greater dependence on the severity of the disease with a more severe disease (e.g. pancreatic cancer) requiring a lower critical value (e.g. *z*_*α*_=0.587,*α*=0.279) and a less severe disease (e.g. prostate cancer) requiring a higher critical value (e.g. *z*_*α*_=2.252, *α*=0.012).

In the example presented above, we made some simplifying assumptions from the Abrahamyan et al. model for tractability, such as assuming independence of costs and clinical outcomes, which lead to slightly different optimal designs. In our model, we assumed that there is a non-trial cost in treating a patient with the experimental treatment, *c*_2_, that is known and fixed. However, *c*_2_ may also be interpreted as the harm and risk of the experimental treatment. Other authors, for example, have interpreted this cost as critical event, treatment for side effects due to the experimental treatment, the loss from the reversal of a decision which is when after the decision to adopt the new treatment is made but following information from subsequent patients the treatment has to be withdrawn, or observing adverse events both within and outside the trial which will affect costs and take-up of the new treatment [18–20]. As such the optimisation of sample size may depend on two endpoints; efficacy and safety. This may be simplified by aggregating efficacy and safety in a clinical utility index that could be used as the primary endpoint. Therefore, the unknown parameter, *θ*, would represent the mean difference in this utility index.

There are two possible decisions in our model where the experimental treatment is approved for treating future patients depending if the observed difference is greater than the optimal threshold. An alternative to defining the threshold at some frequentist test size, we could define it as some monetary value such as willingness to pay for one unit of health, e.g. in the model proposed by Willan and Eckermann where the decision making is from the perspective of the society who is responsible to decide whether or not to reimburse a new intervention at the given price to the company. In their model, the societal’s decision is based on the threshold of the willingness to pay at a certain price [21].

We also assume that the size of the population is known. Based on Abrahamyan et al., who estimated the annual incidence to be 200, we took *N*=4000 corresponding to a 20 year period of market exclusivity (the time during which the new treatment is the sole drug in the market). Alternatively, the population size could be modelled by considering the accrual time, market exclusivity period (the new treatment being the sole drug in the market) and the delay taken from the availability of results to the submission to the regulatory approval, production, marketing, distribution and sales [11, 19, 21, 22]. The inclusion of time or a model of growth and decay in our model is straight forward.

The estimate of the proportion of patients who will benefit from the new treatment if it is approved for routine use may be more complex by including patient’s life expectancy and quality of life [23–25]. Or depending on the severity of the disease, the number of future patients who would take up the recommended new treatment depends on the treatment efficacy in a piecewise linear function [10, 26–29]. There may be no future patients who receive the treatment if the effectiveness is lower than a predefined threshold and some maximum number of patients if it is greater than a predefined threshold, i.e. the improvement is sufficiently large. If the efficacy is in between the lower and upper thresholds, the number of patients is a linear function that increases with the efficacy. This could effectively limit the choice of the optimal value *α*^∗^ as insufficiently strong evidence from the trial of a treatment effect might lead to the new treatment not being used for any patients following the trial.

A challenge associated with implementation of the method proposed is specification of the prior distribution. A number of authors have discussed elicitation of prior distribution parameters for Bayesian trial design, including in the setting of a rare disease [9, 30]. Using a more informative prior generally results in a smaller optimal trial sample size, sometimes with the optimum to be to conduct no trial at all but to make a decision based on prior data alone. If this is considered to be inappropriate, careful consideration should be given to the prior distribution as well as the specification of parameters of the utility function.

We have assumed that regulatory agencies make a simplistic binary decision, approve or not the submitted product. Agencies such as FDA/EMA may also give “accelerated approval/conditional approval”. Our proposed model can be expanded to include this decision where the utility function for this action may depend on the subsequent actions that the sponsor would take. Those subsequent actions may depend on whether or not the regulatory agencies might take given future observations. This type of sequential decision-theoretic model has been explored by various authors [31–35].

## Conclusions

Decision-theoretic VOI analysis provides an alternative to conventional power calculations for the determination of the sample size for a clinical trial. Using such an approach, the final decision at the end of the trial and choice of the trial sample size are made so as to maximise the posterior expected value of a specified utility function.

Although the trial is not designed so as to control frequentist error rates at specified levels, since the decision to approve a new treatment will be taken so long as the observed mean difference is sufficiently large, this is equivalent to a frequentist hypothesis test conducted with a type I error rate corresponding to the optimal decision. The method can thus be seen as providing a flexible approach in which both the type I error rate and the power (or equivalently the sample size) of a trial can reflect the size of the future population for whom the treatment under investigation is intended. We believe such an approach could be particularly valuable in the setting of a small population such as a trial of a rare disease, when there is likely to be considerable knowledge of the size of the target population.

## Additional files

Additional file 1 Derivation of expected utility function. Detailed mathematical derivation of results cited in main paper. (PDF 102 kb)

Additional file 2 Additional figures for different prior distributions. Additional figures similar to Fig. 3 in the main paper giving optimal designs and operating characteristics for a range of prior distribution mean and variance values. (PDF 112 kb)

## Abbreviations

AP: Alternate day prophylaxis
OD: On demand
RCT: Randomised controlled trial
TP: Tailored prophylaxis
VOI: Value of information
