# Supplementary material for: Value of information methods to design a clinical trial in a small population to optimise a health economic utility function
Source: BMC Med Res Methodol. 2018 Feb 8;18:20. doi: 10.1186/s12874-018-0475-0 (PMC5806391; doi:10.1186/s12874-018-0475-0)
Supplement: Supplementary file 1 — Derivation of expected utility function. Detailed mathematical derivation of results cited in main paper. (PDF 102 kb) [file 12874_2018_475_MOESM1_ESM.pdf]

Derivation of expected utility function

Assuming that the difference between means follows a normal distribution, $\bar{X} \sim N(\theta, \tau^2/n)$, and the unknown parameter also follows a normal distribution, $\theta \sim N(\mu_0, \sigma_0^2)$. The optimal utility is

$$G(n,\theta) = (\theta - c_2)(N - n/\rho) I_{\{\bar{x} > z_\alpha \tau/\sqrt{n}\}} + \frac{n}{2}(\theta - c_2) - c_1 n - c_f I_{\{n>0\}}.$$

The expectation of the utility is the expectation taken over the posterior distribution of θ for all possible values of responses, $\bar{X}$. However, as $\bar{X}$ is unknown at the planning stage the expected utility of the optimal decision is obtained from the distribution of $\bar{X}$ given θ and n. As θ is unknown, the expectation of the expected utility is taken over the prior distribution of θ,

$$\begin{aligned}
&\mathcal{G}(n, z_\alpha) \\
&= \iint \big[(\theta - c_2)(N - n/\rho) I_{\{\bar{x} \geq z_\alpha \tau/\sqrt{n}\}} + (\theta - c_2)\frac{n}{2} \\
&\quad - c_1 n - c_f I_{\{n>0\}}\big] f(\theta|\bar{x}) f(\bar{x}|n)\, d\bar{x}\, d\theta \\
&= (N - n) \int_{z_\alpha \tau/\sqrt{n}}^{\infty} \int_{-\infty}^{\infty} \theta f(\theta|\bar{x}) f(\bar{x}|n)\, d\theta\, d\bar{x} \\
&\quad - c_2 \Phi(-Z)(N - n/\rho) + \frac{n}{2}(\mu_0 - c_2) - c_1 n \\
&\quad - c_f I_{\{n>0\}} \\
&= (N - n/\rho)\Big\{(\mu_0 - c_2)\Phi(-Z) + \frac{\sigma_0^2}{\sigma_x}\phi(Z)\Big\} \\
&\qquad + \frac{n}{2}(\mu_0 - c_2) - c_1 n - c_f I_{\{n>0\}} \qquad (1)
\end{aligned}$$

where $f(\theta|\bar{x})$ is the posterior density of θ given $\bar{x}$, $f(\bar{x}|n)$ is the prior predictive density function of $\bar{x}$, $\Phi(\cdot)$ is the normal cumulative distribution function, $\phi(\cdot)$ is the normal density function, $Z = \frac{z_\alpha \tau/\sqrt{n} - \mu_0}{\sigma_x}$ and $\sigma_x^2 = \sigma_0^2 + \tau^2/n$. The expected utility is a function of n and z_α.
