# Supplementary material for: Value of information methods to design a clinical trial in a small population to optimise a health economic utility function
Source: BMC Med Res Methodol. 2018 Feb 8;18:20. doi: 10.1186/s12874-018-0475-0 (PMC5806391; doi:10.1186/s12874-018-0475-0)
Supplement: Supplementary file 2 — Additional figures for different prior distributions. Additional figures similar to Fig. 3 in the main paper giving optimal designs and operating characteristics for a range of prior distribution mean and variance values. (PDF 112 kb) [file 12874_2018_475_MOESM2_ESM.pdf]

Additional figures: Optimal designs and operating characteristics for a range of prior distributions

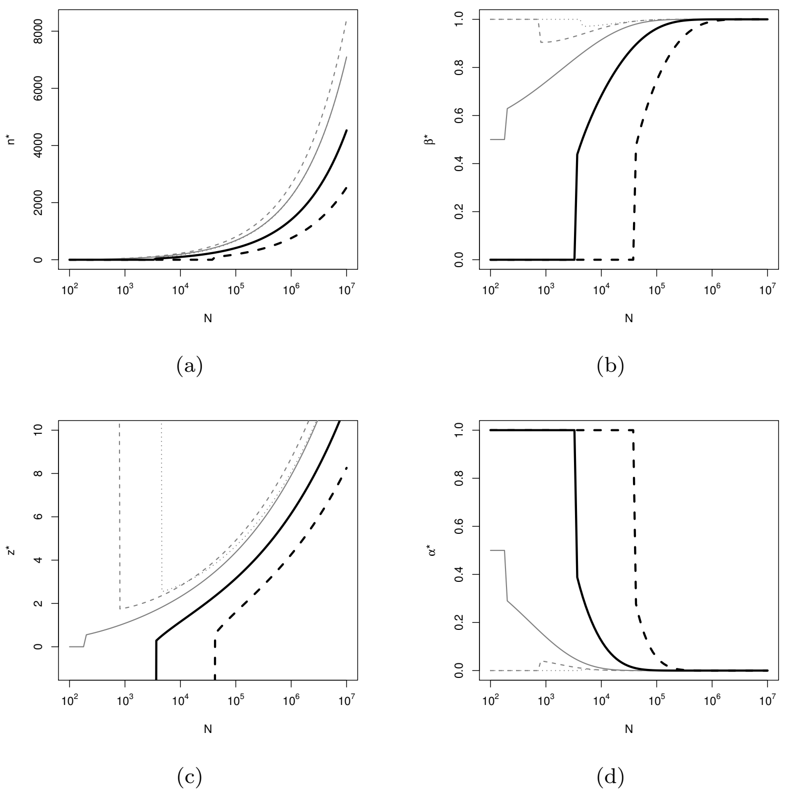

Figure 1: Optimal (a) sample size, n^*, (b) type II error rate, β^*, (c) z^*_α and (d) type I error rate, α^* in order to detect an alternative $\theta_A = \$24819$ against the size of the population, N, with fixed $\sigma_0^2 = (\$49638)^2, \tau^2 = (\$363202)^2, c_1 = \$5000, c_2 = \$61032$ and c_f=\$1 million for different values of; $\mu_0 = 0$ (light grey dotted line), $\mu_0 = \$32000$ (light grey dashed line), $\mu_0 = c_2 = \$61032$ (light grey solid line), $\mu_0 = \$96000$ (heavy black solid line), and $\mu_0 = \$128000$ (heavy black dashed line).

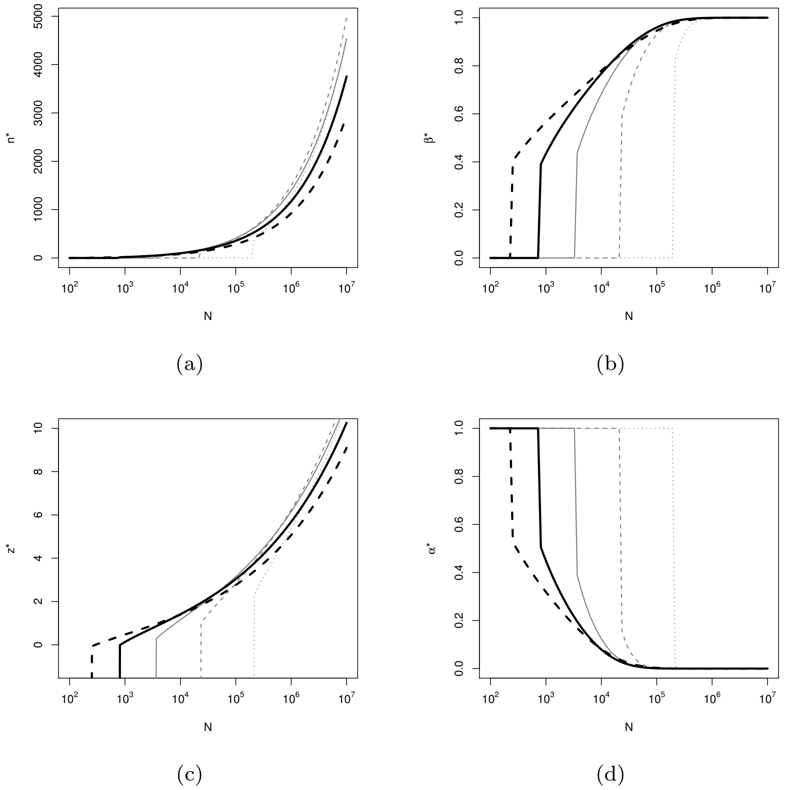

Figure 2: Optimal (a) sample size, n^*, (b) type II error rate, β^*, (c) z_α^* and (d) type I error rate, α^* in order to detect an alternative $\theta_A = \$24819$ against the size of the population, N, with fixed $\mu_0 = \$96000, , \tau^2 = (\$363202)^2, c_1 = \$5000, c_2 = \$61032$ and c_f=\$1 million for different values of; $\sigma_0^2 = (\$49638)^2/4$ (light grey dotted line), $\sigma_0^2 = (\$49638)^2/2$ (light grey dashed line), $\sigma_0^2 = (\$49638)^2$ (light grey solid line), $\sigma_0^2 = 2(\$49638)^2$ (heavy black solid line) and $\sigma_0^2 = 4(\$49638)^2$ (heavy black dashed line).
